# PEOPLE DO NOT AGE IN LABS: SOCIAL AND BEHAVIORAL CONSIDERATIONS WHEN DEVELOPING GEROSCIENCE INTERVENTIONS

**DOI:** 10.1093/geroni/igae098.1341

**Published:** 2024-12-31

**Authors:** Eli Puterman

**Affiliations:** University of British Columbia, Vancouver, British Columbia, Canada

## Abstract

Geroscience researchers are determined to identify the constellation of genetic, molecular, and cellular mechanisms of aging that, eventually, will be translated into anti-aging therapies and pills. It is argued, however, that without a consideration of social and behavioural determinants of health, geroscientists will miss the full potential to impact health and longevity of all humans, not just a select few. Social and behavioural determinants of health are the non-medical, non-biological factors influencing health and disease, and their underlying biological processes. Humans are complicated species, with a lifetime of exposures to stressful, adverse, and sometimes traumatic experiences and a lifetime of engaging in a mix of healthy and unhealthy behaviours. Together, social and behavioural factors shape biological aging through epigenetic embedding and wear and tear of biological systems, causing eventual accelerated biological aging and disease. By understanding what social and behavioural factors are, how they embed biologically and accelerate aging processes, geroscientists can develop a deeper understanding of the complicated nature of human experimental research. Social and behavioural factors impact recruitment, engagement, and adherence of participants in intervention trials, which can obscure significant treatment effects. Social and behavioural factors can also limit treatment effects, when considering how difficult it might be to reverse some of the long-term damage caused by life itself. It is important then, for geroscientists to learn about social and behavioural factors, and the best practices to engage as many people across the wide diversity of life circumstances, to impact the healthspan and lifespan of all.

